# Multimodal MR Features of 8 Cases of Epithelioid Glioblastoma

**DOI:** 10.1155/2020/9586806

**Published:** 2020-10-16

**Authors:** Ji-ping Zhao, Chun-xiao Cui, Jia-chen Wang, Hua-wei Su, Chong-feng Duan, Xue-jun Liu

**Affiliations:** Department of Radiology, The Affiliated Hospital of Qingdao University, Qingdao, China

## Abstract

**Purpose:**

The MRI features of epithelioid glioblastoma (eGBM) were analyzed. The apparent diffusion coefficient (ADC), MR perfusion-weighted imaging (PWI), and magnetic resonance spectroscopy (MRS) findings were quantitatively analyzed.

**Methods:**

The MRI images of 8 cases of eGBM were analyzed retrospectively. The location and edge, signal, peritumoral edema, adjacent meningeal invasion, and enhancement of the lesions were observed. The ADC value, relative cerebral blood volume (rCBV), relative cerebral blood flow (rCBF), and N-acetylaspartate/acetylcholine (NAA/Cho) value were analyzed.

**Results:**

Among the 8 patients, the tumors were mainly located in the temporal lobe (*n* = 3), frontal lobe (*n* = 3), and parietal lobe (*n* = 2). The lesion boundary was clear in 6 cases and unclear in 2. The lesions were superficial in 5 cases and in the deep white matter in 3. Internal hemorrhage was observed in 4 cases. There was cystic necrosis in 7 cases, and only 1 case was solid without cystic necrosis. There was no edema around the lesion in 1 case, severe edema in 5, and moderate edema in 2. In 4 cases, the adjacent meninges were involved, and in 1 case, the ependyma was involved. Two patients developed leptomeningeal metastasis within 2 months after the operation. The average ADC value of the tumor parenchyma among all 8 patients was7.15 × 10^−4^ mm^2^/s,which was 17.6% lower than that of the contralateral side. The Cho/NAA metabolite ratio was 5.27 and 0.81 in the lesions of 2 patients. The rCBV was 3.51 ml/100 g and 3.32 ml/100 g of lesions in 2 patients; these values were 36% and 29% higher, respectively, than those of the contralateral side. The rCBF was 31.5 ml/100 g/min and 82.1 ml/100 g/min of lesions in two patients; these values were 49% and 203% higher, respectively, than those of the contralateral side.

**Conclusion:**

eGBM characteristics include a superficial location, easy cyst degeneration, easy necrosis and hemorrhage, and clear boundaries. It easily invades adjacent meninges and shows cerebrospinal fluid dissemination and metastasis. Combining new MR techniques, such as ADC values, PWI, and MRS, could be helpful for improving diagnostic accuracy.

## 1. Introduction

In 2016, the World Health Organization (WHO) published a new standard for the classification of tumors in the central nervous system[1]. eGBM is a new pathological subtype of glioblastoma, isocitrate dehydrogenase (IDH) wild type, and has a pathological grade of IV. It is a highly invasive tumor [1, 2] that was named mainly based on the finding that a large number of epithelioid and rhabdoid tumor cells can be seen in the tumor tissue. At present, research on the imaging features of eGBM mainly consists of case reports or group cases, and most radiologists are not familiar with its clinical and imaging features. In this study, the clinical and imaging data of 8 patients with eGBM who were admitted to the Affiliated Hospital of Qingdao University from October 2016 to November 2018 were collected, and their imaging features were analyzed to improve understanding of this tumor.

## 2. Materials and Methods

### 2.1. General Information

A total of 8 cases of eGBM were collected. Among these, 6 were male and 2 were female patients. The age of the patients ranged from 27 to 68 years old, with an average of 51 years old. The clinical symptoms, treatment, and prognosis are shown in Table 1. One patient (case 5) had a previous history of resection of intracranial pleomorphic xanthoastrocytoma. All 8 patients underwent plain and enhanced MRI scans (8/8), 2 underwent multivoxel MRS imaging (2/8), and 2 underwent PWI imaging (2/8). At the same time, the ADC values were measured in all 8 patients. The solid part of the tumor was selected as the region of interest and was measured by 3 experienced radiologists. The average ADC value was determined. Eventually, all 8 patients underwent tumor resection, and all were confirmed to be eGBM by pathology.

### 2.2. Inspection Method

A GE3.0T MRI and 32-channel head coil were used. The main scanning sequence and parameters were as follows: T1WI (FSE sequence, TR: 1708 ms, TE: 10 ms, sagittal and axial position, slice thickness: 5.5 mm, spacing: 1.0 mm, NEX: 2), T2WI (fast FSE sequence, TR: 3580 ms, TE: 104 ms, axial position, slice thickness: 5.5 mm, spacing: 1.0 mm, NEX: 2), T2 flair (fast FSE, TR: 8402 ms, TE: 131 ms, axial position, slice thickness: 5.5 mm, spacing: 1.0 mm, NEX: 2), and DWI (EPI-SE, TR =2120 ms, TE =63 ms, *B* values were 0 and 1000 s/mm, axial position, slice thickness: 5.5 mm, spacing: 1.0 mm). Enhanced scanning was performed with Gd-DTPA as the contrast medium. Axial, coronal, and sagittal T1WI scans were performed, and the scanning parameters were the same as those used for plain T1WI.

### 2.3. Image Analysis and Pathological Examination

On MRI, the focus location, edge, and internal signal as well as hemorrhage, peritumoral edema, enhancement characteristics, and meningeal metastasis were observed. Brain edema was measured using the following parameters: the maximum diameter of the front and back and the left and right in the axial plane (*A* and *B*, respectively) and the maximum height of the coronal plane (*C*). A formula (*V* = 4/3*π* × *ABC*) was used to calculate the volume of tissue including the edema and the tumor (*V* edema + *V* tumor) or the tumor volume alone (*V* tumor). The edema index (EI) was calculated as (*V* edema + *V* tumor)/*V* tumor, with EI = 1 indicating no edema, 1 < EI < 1.5 indicating mild edema, 1.5 ≤ EI < 3 indicating moderate edema, and EI ≥ 3 indicating severe edema [3]. HE staining and immunohistochemical examination were performed in all patients after the operation.

### 2.4. Statistical Analysis

Paired sample*t*test was used to analyze the difference of ADC value between lesions and contralateral normal brain parenchyma. When *p* < 0.05, the difference was statistically significant.

## 3. Results

### 3.1. Imaging Findings

**Figure 1:**
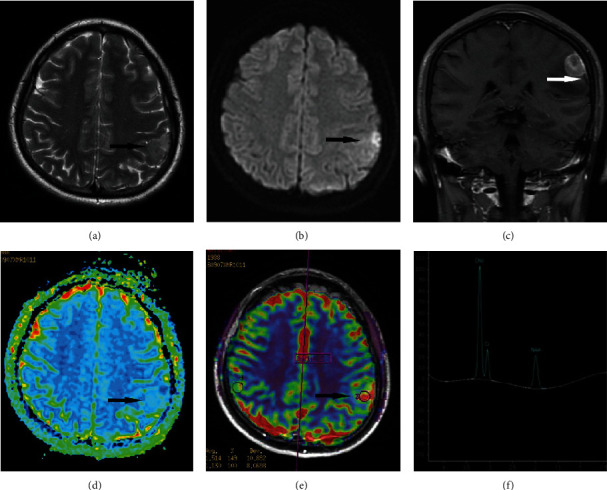
The patient shown was a 27-year-old female patient with eGBM in the left parietal lobe. The lesions involved the cortical and subcortical white matter. The boundary of the lesion was clear. There was no edema around the focus (a, black arrow). T2WI showed a high signal, DWI showed a high signal (b, black arrow), and ADC showed a low signal (d, black arrow). On a contrast-enhanced scan, the lesions showed obvious uniform enhancement, there was no necrosis, the adjacent meninges were involved, and the meningeal tail sign (c, white arrow) could be seen. The tumor showed significantly high perfusion on MR perfusion imaging (e, black arrow), and the rCBF was 31.5 ml/100 g/min, which was 49% higher than that of the contralateral side. According to MRS, the Cho/NAA ratio of the tumor was 5.27 (f).

**Figure 2:**
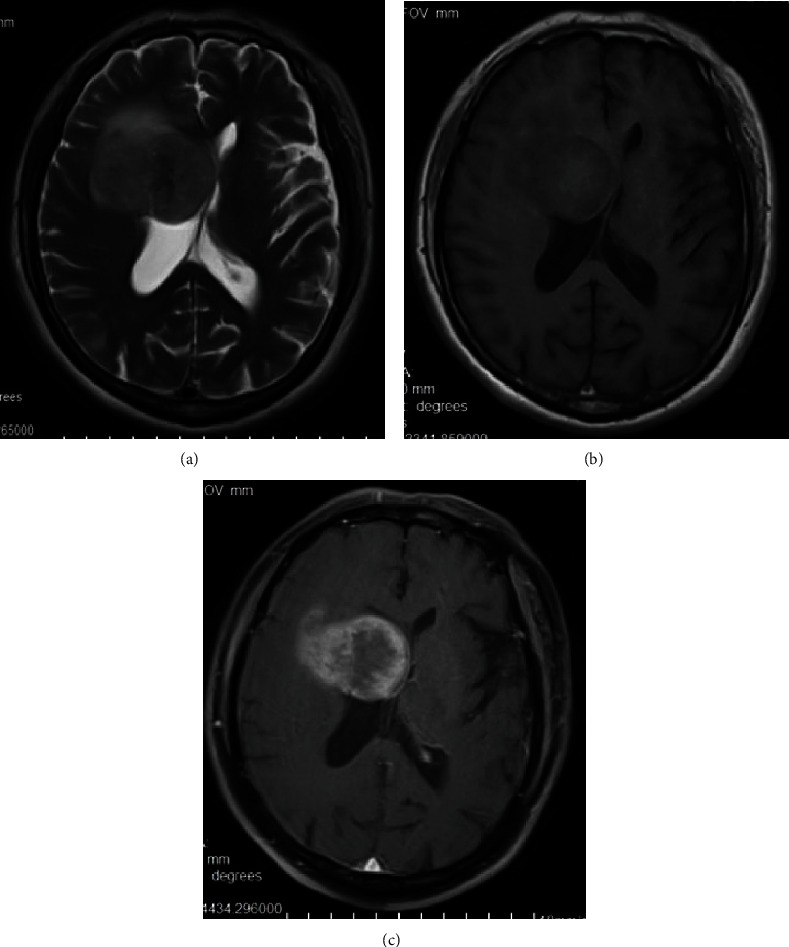
The patient shown was a 66-year-old male patient with eGBM in the deep white matter of the right frontal lobe. Part of the lesion protruded into the right ventricle and showed (a) a slightly high signal on T2WI and (b) an iso-low signal on T1WI. The boundary of the lesion was clear. On a contrast-enhanced scan, the mass showed inhomogeneous enhancement (c).

**Figure 3:**
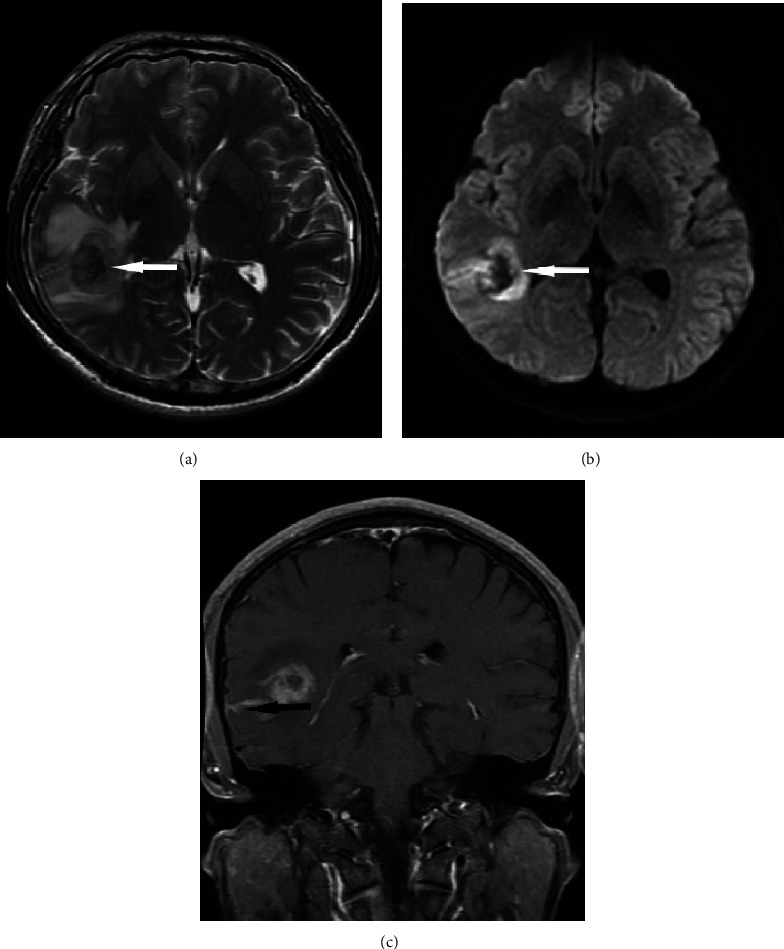
The patient shown was a 41-year-old male patient with white matter eGBM in the right temporal lobe. The boundary of the lesion was unclear, and there was a large area of edema around it. The lesion was accompanied by hemorrhage. The lesion showed a low signal on (a, white arrow) T2WI and (b, white arrow) DWI. On a contrast-enhanced scan, the lesion showed inhomogeneous enhancement and invaded the adjacent meninges (c, black arrow).

**Figure 4:**
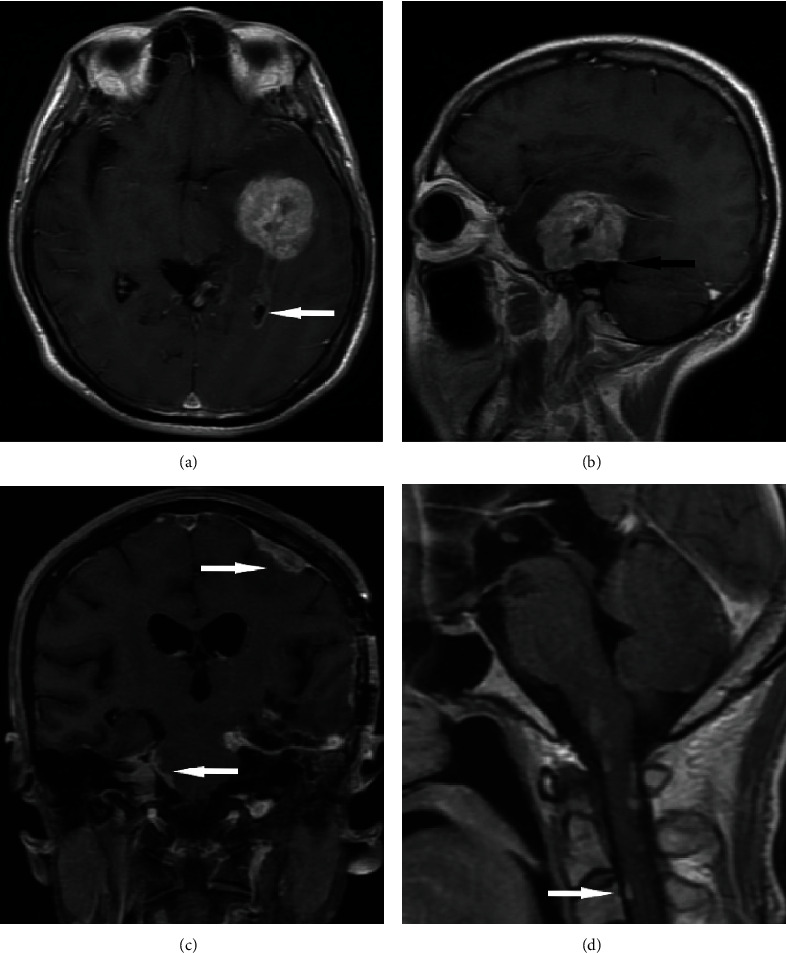
The patient was a 56-year-old male with eGBM in the left temporal lobe. The lesion involved the inferior horn of the left ventricle and showed ependymal metastasis. On a contrast-enhanced scan, the ependyma of the left ventricle showed linear enhancement (a, white arrow). The adjacent meninges were involved, and the meningeal tail sign (b, black arrow) could be seen. Two months after the operation, the tumor showed extensive metastasis had occurred through the cerebrospinal fluid, with multiple sites of thickening and enhancement in the meninges (c, white arrow). Multiple leptomeningeal metastasis found in the cervical spinal cord (d, white arrow).

In these 8 patients, the tumors were located in the temporal lobe (*n* = 3), frontal lobe (*n* = 3), and parietal lobe (*n* = 2). The diameters of the lesions ranged from 2.2 to 5.5 cm, with an average of 4 cm. The lesion boundaries were clear in 6 cases and unclear in 2 cases. The lesions were superficial in 5 cases (Figure 1) and located in the deep white matter in 3 cases, including 1 case in which the tumor was located near and protruded into the ventricle (Figure 2). Hemorrhage was seen in the focus in 4 patients (Figures 3(a) and 3(b)). Cystic degeneration occurred in 3 cases, necrosis in 4 cases, and no cystic degeneration or necrosis in the tumor parenchyma in only 1 case (Figure 1). There was no peritumoral edema in 1 case (Figure 1), severe peritumoral edema in 5 cases (Figure 3), and moderate peritumoral edema in 2 cases (Figure 2). The maximum edema index was 8.1, with an average of 4.24. After enhancement, the solid part of the lesion and the cyst wall were obviously enhanced, but the cystic necrotic area was not. Meningeal involvement and meningeal tail signs were observed in 4 patients on enhanced MRI (Figure 1(c)). One patient also had adjacent ependymal involvement (Figure 4(a)). Leptomeningeal metastasis was found in 2 patients 2 months after the operation (Figure 4(c)), one of them accompanied with cervical spinal cord metastasis (Figure 4(d)). The tumor parenchyma of 5 patients showed a high or slightly high signal on DWI and a low signal on ADC (Figures 1(b) and 1(d)). The tumor parenchyma of 3 patients showed isointense signals on both DWI and ADC. The average ADC value of the tumor parenchyma in these 8 patients was 7.15 × 10^−4^ mm^2^/s, which was 17.6% lower than that of the contralateral side. Paired sample *t* test showed that the difference was statistically significant (*p* < 0.05). MRS showed that the NAA peak decreased significantly, while the Cho peak increased accordingly. The Cho/NAA metabolite ratio was 5.27 and 0.81 in 2 patients (Figure 1(f)), and the average was 3.04. PWI showed that the rCBV of the lesions increased in 2 patients (Figure 2(d)), whose rCBV values were 3.51 and 3.32 ml/100 g (36% and 29% higher, respectively, than those of the contralateral side) and rCBF values were 31.5 and 82.1 ml/100 g/min (Figure 1(e), 49% and 203% higher, respectively, than those of the contralateral side).

### 3.2. Pathological Findings

**Figure 5:**
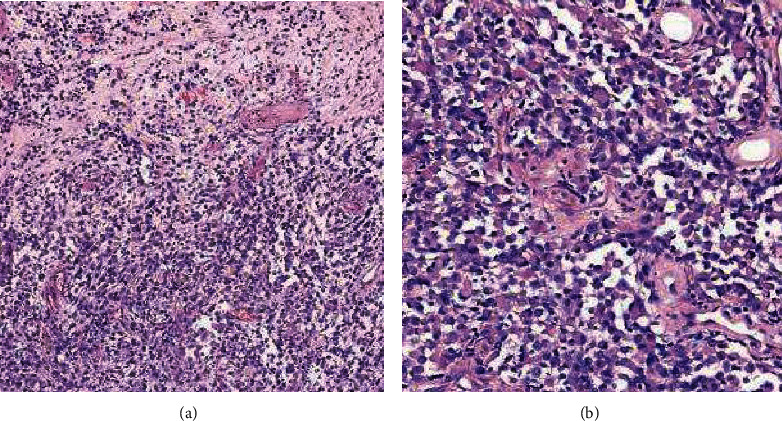
Pathological picture of the patient. HE staining showed that the tumor cells were diffuse and epithelioid tumor cells were dominant. The tumor cells were heteromorphic, eosinophilic in the cytoplasm, nuclear deviation and mitosis. Some areas were epithelioid or rhabdoid. There were multiple necrosis in the stroma. Interstitial vascular proliferation was significant, and capillary endothelial cells showed glomerular proliferation (a: HE ×100; b: HE ×200).

Tumor cells showed diffuse infiltration growth. Some areas were rhabdomyoid. Mitotic figures are visible. Palisade necrosis was seen locally (Figure 5). Immunohistochemistry showed that vimentin, BRAF V600E, ATRX, ini-1, and p53 were positive. The positive rates of S-100, CD34, Olig-2, GFAP, NeuN, and syn were 75%, 62.5%, 50%, 37.5%, and 25%, respectively. Other antibodies idh-1, desmin, myod-1, EMA, myogenin, and LCA were negative. Ki-67 expression was 5%-50%, with an average of 25.5%. Fish showed no chromosome 1 P/19q deletion in 8 cases.

## 4. Discussion

eGBM is a rare type of glioblastoma. Previous cases of pleomorphic xanthoastrocytoma (PXA) that transformed into epithelioid glioblastoma have been reported [4], suggesting that there is a close relationship between these two types of tumors. In our group, there was one patient with eGBM who had a history of surgery for PXA. Most of the reported eGBM patients are young people or children [5], but all the cases in our group were adults, and only 2 were young people. This may be because our hospital is a general hospital to which fewer children come for treatment. The ratio of males to females in this group was 3 : 1, while the male to female ratio was 5 : 2 in a study reported by Huang et al. [6]. This ratio is similar than those of other types of glioblastoma. eGBM lesions are often located in the temporal lobe and frontal lobe [5, 6]. Consistent with these results, in our group, the lesions were mainly located in the frontal lobe and temporal lobe, followed by the parietal lobe. Most tumors in this group were located superficially, essentially consistent with a report by Huang et al. [6]. Therefore, compared with other intracranial gliomas, eGBM lesions have a superficial position as one of their main features.

### 4.1. MRI Manifestations

*MR signal characteristics of the lesions*: previous studies have shown that the radiologic characteristics of eGBM are usually similar to those of low-grade gliomas, such as pilocytic astrocytoma, PXA, and ganglioglioma [7–9]. Cystic degeneration and nodular enhancement are often found in these lesions. Moreover, eGBM also shows characteristics of malignant tumors [10], such as obvious central necrosis and corolla-like enhancement. In our group, 37.5% (3/8) of the cases showed obvious cystic degeneration, whereas typical pleomorphic glioblastoma rarely shows cystic degeneration; 50% (4/8) of the lesions showed necrosis, which could make eGBM difficult to distinguish from pleomorphic glioblastoma. Intratumoral hemorrhage was seen in some of the eGBM lesions [11]. In this group, 37.5% (3/8) of the lesions showed a hemorrhage signal, similar to previous reports in the literature.

*The edge of focus and the perifocal edema*: the boundaries of most eGBM lesions are clear [6]. In our group, the lesion boundary was clear in 75% (6/8) of the cases, consistent with reports in the literature. In addition, severe edema was observed around the lesion in 62.5% (5/8) of the cases, consistent with a literature report [1]. However, some studies have reported that the edema around the tumor is mild in eGBM [3, 6]. In our group of cases, 1 case showed no edema around the focus. We speculate that tumor edema may be related to the growth process of the disease, with the early stage showing no cystic degeneration or necrosis and therefore only mild edema around the focus, and later growth of the tumor associated with the appearance of cystic degeneration and necrosis and consequently increased peripheral edema.

*Adjacent meningeal invasion and cerebrospinal fluid dissemination*: in this group of cases, 50% (4/8) of the lesions showed adjacent meningeal invasion and the “meningeal tail sign” after enhancement. Huang et al. [6] reported that 2 of 7 patients showed meningeal tail signs, whereas other brain parenchymal tumors had a lower chance of showing meningeal tail sign; this could therefore be an important difference between eGBM and other brain parenchymal tumors. In addition to the invasion of the adjacent meninges, 2 cases recurred within a short time after the operation, resulting in multiple leptomeningeal metastases through cerebrospinal fluid. It has been reported that leptomeningeal spread and metastasis occur in the early stage of eGBM [12], and this is an important point that distinguishes eGBM from typical pleomorphic glioblastoma. The reason for this difference may be that the expression of the BRAF gene activates the extracellular signal-regulated kinase Erk1/2, leading to cell diffusion [13]. Li et al. [14] reported the pathological features of 13 cases of eGBM. They found that meningeal and/or spinal meningeal metastasis occurred in 9 cases (69.7%). One patient in this group showed both ependymal and adjacent meningeal invasion before the operation, this was rare in previous reports, and it was likely to have been because the location of eGBM was generally superficial, and the probability of ependymal involvement was therefore low.

The ADC value can predict the grade of glioma, guide treatment, and predict prognosis [15]. The ADC values of the solid parts of the lesions in this group were lower than those of the contralateral side, indicating that the tumor cells were dense and the degree of malignancy was high [16]. In this group, the Cho/NAA ratio was significantly increased in 2 cases, and the decrease in the NAA peak observed in these cases indicated the destruction of neurons, while the increase in the Cho peak indicated the active proliferation of tumor cells [17]. An increase in the rCBF value indicates that a tumor has an abundant blood supply and is undergoing active tumor cell proliferation [18]. In our group, rCBV and rCBF were significantly higher than normal levels in 2 cases, indicating a high degree of malignancy. Although these multimodal MR parameters cannot determine the pathological classification of glioma, the above parameters strongly suggest that these lesions are highly malignant tumors, which provides a quantitative basis for the preoperative grading to tumors.

### 4.2. Differential Diagnosis

eGBM should be differentiated from pleomorphic glioblastoma, pleomorphic xanthoastrocytoma, pilocytic astrocytoma, metastatic tumor, etc. Pleomorphic glioblastoma usually occurs in patients aged 45 to 75 years old and is usually located in the deep white matter, while the solid part of the pleomorphic glioblastoma usually shows corolla-like enhancement on contrast-enhanced scans. Pleomorphic xanthoastrocytoma usually occurs in children and adolescents and is especially common in the temporal lobe. Its typical manifestation is cystic degeneration with mural nodules and mild peripheral edema [19]. Pilocytic astrocytoma usually occurs in teenagers under 20 years old, especially in the infratentorial and midline areas, and shows mild peritumoral edema. The typical imaging manifestation of this condition is “a small nodule in a large cyst.” Because the location of eGBM is superficial, the boundary of the tumor is clear and the surrounding edema is obvious, and it is therefore easily confused with metastatic tumors. However, metastatic tumors are more common in elderly individuals than in younger populations, and most affected patients have a clear history of primary tumors [1].

### 4.3. Treatment and Prognosis

The prognosis of eGBM is very poor. Most of these tumors grow rapidly and invade the adjacent leptomeninges in the early stage. The tumor cells easily spread through the cerebrospinal fluid. Some studies have shown that the incidence of metastasis of eGBM is as high as 69.7% [14]. It also frequently recurs after operation. In our group, 2 of 8 patients showed recurrence with leptomeningeal metastases along the cerebrospinal fluid within a short time after the operation, and one of them accompanied with cervical spinal cord metastasis. The probability of metastasis along the cerebrospinal fluid is very low in other types of GBM, and patients therefore are usually only examined on brain MRI. However, because eGBM is a special type of GBM, spinal cord enhancement MR should be performed both before an operation and during postoperative follow-up in affected patients. When spinal cord leptomeningeal metastasis is found, radiotherapy of the whole central nervous system should be performed [14]. Unfortunately, even after total resection of the tumor and both radiotherapy and chemotherapy, most patients will still die within 1 year [20].

## 5. Conclusion

The MRI manifestations of eGBM include some specific characteristics, such as a superficial location, frequent cystic degeneration, necrosis, hemorrhage, a clear boundary, frequent involvement of the adjacent meninges, and frequent spread and metastasis through the cerebrospinal fluid. However, in most cases, it is difficult to distinguish eGBM from other malignant brain tumors. ADC values, PWI, MRS, and new MR technology cannot identify the type of tumor cases, but it is helpful for preoperative tumor grading. Because eGBM can easily invade the adjacent meninges and spread through the cerebrospinal fluid, the prognosis of eGBM is very poor. Its treatment is different from that of ordinary high-grade gliomas. As radiologists, we should carefully analyze each image feature, combining these new MR techniques, to make a more accurate preoperative diagnosis, which can help clinicians choose the best treatment plan.

## Figures and Tables

**Table 1:** Clinical characteristics of 8 cases of epithelioid glioblastoma.

Case	Gender	Age (years)	Symptom	Position	Tumor size	Postoperative treatment	Prognosis
1	Female	27	Paroxysmal aphasia with convulsions	Left parietal cortex and subcortical	2.3 cm × 2.0 cm	Postoperative radiotherapy and chemotherapy	Died 12 months after operation
2	Male	53	Headache with drowsiness	Deep white matter of right temporal lobe and insula	6.7 cm × 4.4 cm	Radiotherapy was performed 2 weeks after operation	Died 18 months after operation
3	Male	41	Intermittent headache	Deep white matter of right temporal lobe	3.0 cm × 2.7 cm	No radiotherapy or chemotherapy	Died 7 months after operation
4	Male	56	Dizziness and blurred vision	Left temporal cortex and white matter	4.8 cm × 4.0 cm	Radiotherapy was performed 2 weeks after operation	Died 6 months after operation
5	Female	68	Language barrier	Left frontal cortex and subcortical white matter	6.2 cm × 4.2 cm	Radiotherapy was performed 2 weeks after operation	Survived 3 years after operation until now
6	Male	32	Paroxysmal limb convulsion and stiffness	Left subcortical white matter of frontal lobe	2.0 cm × 3.0 cm	Postoperative radiotherapy and chemotherapy	Died 11 months after operation
7	Male	66	Persistent headache	Deep white matter of right frontal lobe	4.0 cm × 5.0 cm	No radiotherapy or chemotherapy	Died 11 months after operation
8	Male	66	Headache	Subcortical white matter of left parietal lobe	4.2 cm × 5.4 cm	No radiotherapy or chemotherapy	Died 10 months after operation

## Data Availability

All data are in the manuscript.

## Abbreviations

eGBM: Epithelioid glioblastoma
PWI: Perfusion-weighted imaging
MRS: Magnetic resonance spectroscopy
rCBV: Relative cerebral blood volume
rCBF: Relative cerebral blood flow
NAA/Cho: N-Acetylaspartate/acetylcholine
IDH: Isocitrate dehydrogenase
PXA: Pleomorphic xanthoastrocytoma.
